# Health-Promoting Behaviours following Primary Treatment for Cancer: A Rural–Urban Comparison from a Cross-Sectional Study

**DOI:** 10.3390/curroncol30020122

**Published:** 2023-01-25

**Authors:** David Nelson, Ian McGonagle, Christine Jackson, Trish Tsuro, Emily Scott, Mark Gussy, Ros Kane

**Affiliations:** 1 Lincoln International Institute for Rural Health, College of Social Science, University of Lincoln, Lincoln LN6 7TS, UK; 2 Macmillan Cancer Support, London SE1 7UQ, UK; 3 School of Health and Social Care, College of Social Science, University of Lincoln, Lincoln LN6 7TS, UK; 4 United Lincolnshire Hospitals NHS Trust, Research and Innovation Department, Pilgrim Hospital, Boston PE21 9QS, UK; 5 Lincolnshire Partnership NHS Foundation Trust, Peter Hodgkinson Centre, Lincoln County Hospital, Lincoln LN2 5UA, UK; 6 La Trobe Rural Health School, College of Science, Health and Engineering, La Trobe University, Bendigo P.O. Box 199, Australia

**Keywords:** rural health, urban health, cancer survivorship, oncology, health behaviours, health promotion, living with cancer, rural–urban, United Kingdom

## Abstract

Aim: To compare health-promoting behaviours among rural and urban residents following primary treatment for cancer. Methods: A cross-sectional survey collecting demographic variables and data pertaining to health-promoting behaviours, documented using the 52-item Health Promotion Lifestyle Profile II (HPLP-II) measure, which is categorised into six subscales: (1) health responsibility, (2) spiritual growth, (3) physical activity, (4) interpersonal relations, (5) nutrition, and (6) stress management. Residence was defined using the U.K. Office for National Statistics RUC 2011 Rural Urban Classifications. The Index of Multiple Deprivation (IMD) Decile was used to measure deprivation. Quantitative data were analysed using independent samples *t*-test and multiple linear regression. Qualitative data from open-ended questions were analysed thematically. Results: In total, 227 participants with a range of cancer types completed the questionnaire. Fifty-three percent were residents in urban areas and forty-five percent in rural areas. Rural participants scored significantly higher on health responsibility (*p* = 0.001), nutrition (*p* = 0.001), spiritual growth (*p* = 0.004), and interpersonal relationships (*p* = 0.001), as well as on the overall HPLP-II (*p* = 0.001). When controlling for deprivation, age, marital status, and education, rural–urban residence was a significant predictor of exhibiting health-promoting behaviours. A central theme from the qualitative data was the concept of “moving on” from cancer following treatment, by making adjustments to physical, social, psychological, spiritual, and emotional wellbeing. Conclusions: This research revealed, for the first time, differences in health-promoting behaviours among rural and urban U.K. populations who have completed primary cancer treatment. Rural residence can provide a positive environment for engaging with health-promoting behaviours following a cancer diagnosis and treatment.

## 1. Introduction 

Much of the extant literature from high-income countries points to higher mortality and poorer long-term survival for people living with cancer in rural compared with urban areas [1]. People living with cancer from rural areas have unique unmet psychosocial needs that relate to their rural residence [2]. These needs pertain to travel, access to care, and a higher level of financial problems associated with their diagnosis and treatment when compared with those from urban areas [2,3]. Additionally, it has been documented that rural areas are often underrepresented in cancer research [4]. The cancer survivorship studies that do exist have largely been conducted in North America and Australia [1,2,3,4,5,6,7,8,9,10,11], although there is now a small but growing body of evidence with U.K. populations [12,13,14,15]. What constitutes ‘rural’ in the United Kingdom is arguably different when compared with much larger North American or Australian settings, and definitions of rurality have changed over time and become increasingly complex [16]. Therefore, it cannot be assumed that the existing evidence of international rural disparities elsewhere would easily translate to the U.K. setting. Recent research with people living with prostate cancer in the United Kingdom found that the impact of deprivation and rurality on health-related quality of life was not greater than would be expected in the general population [17]. Other research has shown that people living with cancer from rural parts of the United Kingdom have greater engagement with self-management and higher self-reported health status when compared with urban counterparts [12,13,15]. 

Despite the scientific literature leaning heavily towards the negative health aspects and challenges of rural life, it should be noted that there are several health benefits to rurality that can positively influence the health and wellbeing of people living with and affected by cancer. For example, community support might be more prevalent in some rural areas and rural areas often have good access to green spaces, which can be beneficial to both physical and mental health [13,18,19]. That said, rural communities are not homogenous and rural health disparities require solutions that are tailored and in line with the needs of the local community [20]. Following the completion of primary treatment, people living with cancer are increasingly expected to self-manage the physical, emotional, and social consequences of cancer [21], but research has shown that rural people living with cancer are not always provided with consistent and good quality information to support self-management and facilitate their recovery [11]. Practising health-promoting behaviours can support people living with cancer in their recovery and recent research suggests that rural people living with cancer have information needs in relation to engaging with health-promoting behaviours [22]. These needs pertain to diet and nutrition, physical activity, losing weight or maintaining a healthy weight and decreasing the risk of cancer recurrence. This current research aimed to investigate and compare health-promoting behaviours in a sample of people living with cancer who had completed primary treatment from rural and urban areas in the United Kingdom. 

## 2. Methods

### 2.1. Setting and Design

This cross-sectional study was conducted in the East Midlands region of the United Kingdom, which has been described by researchers as a microcosm in terms of the demographics, rural–urban dynamics, and deprivation, making it a well-suited setting for this study [23]. Participants had been treated at two acute National Health Service (NHS) Trusts in the East Midlands, one that covers a large and sparsely populated area and one located in a city serving a high proportion of urban dwellers. The data used in this article came from a larger mixed methods study that is reported on elsewhere [12,13,15]. The study collected data on demographics and health-promoting behaviours via a self-completion postal questionnaire between June 2017 and February 2018. Completing and returning the questionnaire implied informed consent. Ethics approval was obtained from a National Health Service Research Ethics Committee and the Health Research Authority (IRAS ID: 204679; Ref: 17/WS/0054). This study adhered to the Strengthening the Reporting of Observational studies in Epidemiology (STROBE) guideline for cross-sectional studies [24].

### 2.2. Participants

Participants were recruited on behalf of the research team via two acute cancer centres at the two participating NHS trusts. Both Cancer Centre Managers and Lead Cancer Nurse Specialists at each trust were briefed on the participant eligibility criteria and confirmed that they could identify and recruit potential participants via their patient database on behalf of the research team. Participants were eligible if they were ≥18, had a confirmed cancer diagnosis, had undergone primary cancer treatment in the last five years, and were no longer in receipt of active treatment and registered on the patient database at the two participating NHS trusts. Participants were excluded if they were <18, had evidence of cancer recurrence/metastatic spread, had started active oncology treatment within the last twelve months, or were being treated for palliative or end of life care. There were no restrictions on cancer type. The sample size was determined via a power calculation using the software package Minitab (Version 17, Pennsylvania State University, PA, USA). The calculation allowed for a 20 percent difference between scores and a test with 95 percent power giving a required sample of 417. In line with similar research in the West Midlands of England, it was estimated that 50 percent of participants would respond [25]; therefore, the survey was sent to a random sample of 834 eligible participants (417 at each trust). A random sample was used to ensure that our final dataset was representative of the eligible population and encapsulated a range of different demographics and diagnoses among all of those who met the pre-determined eligibility criteria outlined above. No weighting techniques were used. Additionally, no financial incentives were used to motivate potential participants to take part. Finally, a reminder letter was also not used following ethical review as the ethics committee deemed this inappropriate for this study as it might overburden or cause distress to people trying to recover from cancer. 

### 2.3. Rural–Urban Classifications—RUC 2011 

Rural and urban residence of participants was defined using the U.K. Office for National Statistics (ONS) RUC 2011 Rural Urban Classifications [26]. Respondents were asked for their postcode on the questionnaire and the online ONS postcode look-up tool (https://onsdigital.github.io/postcode-lookup/) was used to assign them to a rural or urban area. The use of the RUC 2011 is advocated for the purposes of statistical analyses by the U.K. Department for Environment, Food, and Rural Affairs [27]. The use of official statistics to define and measure rural–urban areas is also in keeping with methodological approaches used in other cancer survivorship research [28,29,30]. Two percent of participants (*n* = 4) failed to provide their postcode on the questionnaire, so these data were excluded from the rural–urban analysis reported on in this paper.

### 2.4. Measuring Deprivation—The U.K. Decile of Index of Multiple Deprivation

The U.K. decile of Index of Multiple Deprivation (IMD) was used to measure deprivation [31]. This accounts for material deprivation as well as other components such as health, education, and crime [32].

### 2.5. Health-Promoting Behaviours—HPLP-II

The Health Promotion Lifestyle Profile II (HPLP-II) was used to measure health-promoting behaviours [33]. It consists of fifty-two items and is categorised into six health-promoting subscales: (1) health responsibility—9 items, (2) spiritual growth—9 items, (3) physical activity—8 items, (4) interpersonal relations—9 items, (5) nutrition—9 items, and (6) stress management—8 items. It asks respondents to indicate how often they practise specific health-promoting behaviours or wellbeing habits on a fixed four-point Likert scale, where “never” was coded as 1, “sometimes” as 2, “often” as 3, and “routinely” as 4. For example, how often do they “Follow a planned exercise programme”, “Take some time for relaxation”, “Eat 3–5 servings of vegetables a day”, “Use specific methods to control stress”, and “Attend educational programmes on personal health care.” A mean score is calculated for all 52 items, giving the overall health-promoting lifestyle, as well as for each of the six subscales. Where data were incomplete on any of the individual questions on the overall HPLP-II or on the six subscales, this was classed as missing and the mean was not computed for that particular participant, and it was excluded from the final analysis. The use of means rather than sums of scale items was used to retain the 1 to 4 metric of item responses and to allow for meaningful comparisons of scores across the individual subscales. In this study, reliability for the total HPLP-II was high, with a Cronbach’s α of 0.94, and α ranged from 0.72 to 0.88 for the six subscales, indicating good reliability. 

### 2.6. Qualitative Comments

Researchers frequently use open-ended questions at the end of quantitative questionnaires that invite participants to add, in their own words, further information about issues covered in the questionnaire [34,35]. At the end of our questionnaire, respondents were asked if they had any further experiences they wanted to share and these data were analysed thematically [36]. Data were firstly analysed independently by two clinical academic nurses (T.T. and E.S.) who were somewhat less familiar with the theoretical aspects of cancer survivorship in that they did not normally work with or conduct research with cancer survivors, while the other two analysts (I.McG. and D.N.) both had experience in conducting research in this area and were co-investigators on the wider study from which these data were drawn [9,15]. Therefore, the process of analysis took a hybrid approach that used both deductive and inductive approaches to the thematic analysis [37]. The free text data were collected to generate additional insight into the lived experiences of rural and urban cancer survivors and to offer participants the opportunity to add anything else in their own words that they felt would be of importance. 

### 2.7. Statistical Analyses

Data were analysed using IBM SPSS software (Version 27, Chicago, IL, USA). Descriptive statistics were used to characterise the data on demographics and health behaviours. Frequencies, percentages, mean values, and standard deviations were reported. To check for normality, the Shapiro–Wilk test was used. The independent samples *t*-test was used to assess whether there was a statistically significant difference between urban and rural respondents in relation to the mean values on the overall HPLP-II and the six subscales. The *t* value is presented to show the strength of association and mean differences and 95% confidence intervals are reported. To identify confounding variables on rural–urban residence, Pearson’s *r* and Pearson’s chi-square (*χ*^2^) tests were run. Additional confounders such as age and deprivation were identified from the extant literature [4,38,39,40]. Multivariate analysis was conducted using linear regression to ascertain the effect of rural–urban residence on the HPLP-II while controlling for independent confounding variables. The socio-demographic variables included in the modelling were as follows: age (measured in years), marital status (coded as 0 = widowed/single/divorced and 1 = married/civil partnership) and education (coded as 0 = lower than degree and 1 = degree or higher), as well as IMD (coded as 1 = 10% most deprived to 10 = 10% least deprived) and the RUC 2011 (coded as 0 = urban; 1 = rural). Given that multiple comparisons were conducted on a range of outcomes, the threshold for statistical significance was set at *p* < 0.01.

## 3. Results

### 3.1. Participant Characteristics

In total, 227 participants completed a questionnaire (27% response rate). The mean age was 66.86 ± 11.22 (range 26–90). Fifty-two percent (*n* = 119) were female and forty-eight percent (*n* = 108) were male. There were a range of different cancer diagnoses, but the most common were breast (*n* = 73), urological (*n* = 53), and upper and lower gastrointestinal (*n* = 41). Fifty-three percent were resident in an urban area (*n* = 120), forty-five percent were resident in rural areas (*n* = 103), and two percent were unknown residence (*n* = 2) owing to missing postcode data. Full demographic data of respondents are reported on elsewhere [13].

### 3.2. HPLP-II Mean Scores

The mean value for the total HPLP-II was 2.55 ± 0.46 (range 1.38–4.00). Out of the six subscales, respondents scored highest in interpersonal relations with a mean value of 2.94 ± 0.58 (range 1.11–4.00) and in nutrition with a mean of 2.73 ± 0.59 (range 1.00–4.00). They scored lowest in physical activity with a mean value of 2.08 ± 0.73 (range 1.00–4.00), as well as health responsibility with a mean of 2.16 ± 0.53. Table 1 shows the mean scores for the overall HPLP-II and the six subscales as well as the percentage of missing data. Interestingly, there were considerably more rural respondents who did not answer all of the physical activity questions compared with urban. With regard to questions on nutrition, there were more missing data from urban respondents when compared with rural. For the remainder of the questions, the split between rural and urban in terms of missingness was relatively even.

### 3.3. IMD Decile Scores

Table 2 reports on the full IMD Decile scores for rural and urban participants as well as the sample as a whole. There were more urban participants living in the most deprived locations compared with rural, with eighteen percent (*n* = 22) of all urban participants belonging to either decile 1 or decile 2 of deprivation. Conversely, there no rural participants in our sample that had a postcode belonging to decile 1 or decile 2 of the IMD.

### 3.4. HPLP-II Rural–Urban Comparison 

Rural participants scored significantly higher than those living in urban areas with regard to health responsibility (*p* = 0.001), nutrition (*p* = 0.001), spiritual growth (*p* = 0.004), and interpersonal relationships (*p* = 0.001). Rural participants engaged more with physical activity, although this was not significant at *p* < 0.01. There were no significant differences between rural and urban residents relating to stress management. In terms of the overall HPLP-II, rural participants scored significantly higher (*p* = 0.001) than urban participants. Full rural–urban comparisons of the HPLP-II can be found in Table 3.

A multiple linear regression was calculated to ascertain the effect of rural–urban residency while controlling for potential confounders (see Table 4). At *p* < 0.01, Pearson’s chi square (χ^2^) test revealed that there were significant associations between rural–urban residence and living arrangement (*p* = 0.002), marital status (*p* = 0.001), and education (*p* = 0.003). Therefore, these were all included in the multivariate analysis with the exception of living arrangement, as we tested for multicollinearity and the VIF and tolerance statistic suggested collinearity between living arrangement and marital status. Consequently, the decision was made to include just one of these, marital status, in the final modelling. Pearson’s r did not detect any significant association with age (*p* = 0.620) and rural–urban residency, although this was still included in the regression model because of its association with rural and urban populations in the extant literature [4,38]. Finally, deprivation using the IMD was also included in the model because of its strong relationship with health outcomes in both rural and urban areas [39,40].

When controlling for deprivation, age, marital status, and education, rural–urban residence was a significant predictor of health-promoting behaviours. The model as a whole explained twenty percent of the variance (adjusted R_2_ = 0.20, F (5148) = 8.577, *p* < 0.001). At *p* < 0.01, there were no other significant predictors of HPLP-II in this model. In subsequent modelling of the six subscales of HPLP-II, rural–urban residence was also a significant predictor at *p* < 0.01 of behaviours in relation to nutrition (*p* = 0.007) and interpersonal relations (*p* = 0.008) while adjusting for the same confounding variables as above. At *p* < 0.01, IMD was also a significant predictor of nutritional behaviours (*p* = 0.006) in the adjusted analysis. Age was a significant predictor at *p* < 0.01 of behaviours in relation to health responsibility (*p* = 0.001) and physical activity (*p* = 0.001) in the subsequent modelling of the HPLP-II subscales. Finally, marital status was a significant predictor at *p* < 0.01 of interpersonal relations behaviours (*p* = 0.002) while adjusting for deprivation, age, marital status, and education.

### 3.5. Qualitative Results

Fifty-six percent (*n* = 128) of the total sample completed free-text responses at the end of the questionnaire and four themes were identified in the analysis: (1) the idea of “moving on”, (2) “good fortune”, (3) self-management, and (4) support.

*The Idea of “Moving On”*

The main overarching theme from the qualitative data was the concept of “moving on” from cancer following the completion of primary treatment. For most, this incorporated making adjustments to their physical, social, psychological, spiritual, and emotional wellbeing. The idea of “recovery” from cancer was prominent and this incorporated both clinical and personal recovery. Clinical pertains to being free of symptoms and side effects, no longer receiving treatment or follow-up care, as well as being in “remission”. The personal relates to the individual “moving on” and building a new life for themselves after their cancer experience. A participant made the following comment:

*“I was lucky that my surgery completely removed my tumour. Since then, I feel the best medicine for me is to put it behind me.”*

**Female, Urological Cancer, Resident in a Rural Area.**

*“Good Fortune”*

Another theme from the analysis of the data was “good fortune”. Participants gave an account of their outlook for the future, detailing both positivity about “moving on”, together with inevitable apprehensions about the future. Aside from this, many participants stated that they were “glad to still be alive” and they would “count my blessings” and “consider myself very lucky”, and this sense of good fortune in turn influenced their desire to pursue a healthy lifestyle for the future, as evidenced below:

*“As I have been lucky to survive lung cancer, I do treat my life with more respect and try to eat sensibly and take regular exercise to ensure I stay as fit as possible. The 6 monthly checks I receive are very important in ensuring I remain cancer free, and I am so grateful that the monitoring lasts for 5 years. Ideally, I would like the checks to go on longer for added confidence.”*

**Male, Lung Cancer, Resident in an Urban Area.**

*Self-Management*

The behavioural aspects of self-management encompass adjustments made by participants to health behaviours such as leading an active lifestyle, increasing exercise, and making changes to their diet and nutrition. The psychological aspect of self-management encompasses participants building their emotional resilience and strategies for this (e.g., meditation and mindfulness), their emotions and attitudes towards their cancer experience including their outlook on life after cancer, and their attitudes towards managing their health and health care. The participant below explains how activities such as yoga and meditation as well as a meat-free diet have enhanced her recovery:

*“I have started and maintained a holistic and natural lifestyle, this has been through my own research, reading and the internet. I think that a holistic approach is a good way of feeling like you have regained control of your life. I think that things like diet, meditation, yoga etc. should be promoted much more by the cancer care team. Reflexology (Privately) during my treatment also helped me to manage side effects (physical) and also, Reiki has helped the mental side of recovery. I now have a gluten and dairy free lifestyle without meat and concentrate heavily on nutrition.”*

**Female, Gynaecological Cancer, Resident in a Rural Area**.

However, not all participants reported positive experiences of self-management; the below respondent explains how they are having difficulty with pain, fatigue, and emotional management:

*“In constant pain. Acute fatigue. Psychological after effects terrible. Still struggle to cope.”*

**Female, Breast Cancer, Resident in an Urban Area**

*Clinical and Non-Clinical Support*

The final theme incorporates participants identifying their main sources of clinical and non-clinical support, where some of the participants stated they “could not have done it without them” and, in some cases, participants reported they subjectively feel they owe their life to them. Participants reported both positive and negative experiences of clinical support, as evidence below:

*“[Name removed] hospital have been great from diagnosis to now ongoing follow-ups.*

*Many thanks to my GP at [name removed] for pushing for my diagnosis. Great work.*

*God Bless You All x”*

**Male, Lower Gastrointestinal Cancer, Resident in a Rural Area.**

*“I think that because the operation to have a mastectomy and immediate aftercare in the hospital was so poor it has seriously knocked my confidence and most of the time I feel isolated and helpless. No one understands how difficult I am finding coping with everyday life. I should have another operation, but keep putting it off because my original experience was so bad. I can’t face going through it again and feel trapped.”*

**Female, Breast Cancer, Resident in a Rural Area.**

Non-clinical support from friends and family was an important factor for many of the participants. The respondent below explains how they were disappointed with the lack of gender-specific support for males and how they felt family support was crucial to recovery; he stated the following:

*“Was treated with respect–continues with reviews attended Macmillan survivors course–6 weeks which was mostly positive–disappointed by unavailability of men only groups. Family support most important aspect of recovery.”*

**Male, Head and Neck Cancer, Resident in a Rural Area**

Furthermore, these respondents explain how they have a supportive network of non-clinical support through their family and friends:

*“My attitude is very positive and am lucky to have a supportive family (though not nearby) and lots of friends who are and I have been open with them all about my situation. We all know no one lives forever–c’est la vie!”*

**Female, Breast Cancer, Resident in a Rural Area**

*“Have tried hard to continue my way of life, not allowing side effects to ruin my life.*

*Have many family and friends in support.”*

**Male, Urological Cancer, Resident in an Urban Area**

## 4. Discussion 

This research revealed for the first time that health-promoting behaviours significantly differ among rural and urban U.K. people living with cancer who have completed primary treatment. The wider health sciences literature is often weakened by the omission of parallel comparison groups [41] and a particular strength of this study was the good split of rural (*n* = 103) versus urban (*n* = 120) participants in the final sample. There is a need to understand the experiences of people living with cancer from both rural and urban environments, given almost a fifth of the United Kingdom’s total population reside in areas classed as ‘rural’ [42]. It has been reported that rural areas are often underrepresented in cancer research [4] and the cancer survivorship studies that do exist tend to come from North America and Australia [1,2,3,4,5,6,7,8,9,10,11]. Therefore, this research is an important addition to the small but increasing body of U.K. evidence that highlights that people living with cancer from rural areas can have higher cancer-related self-efficacy, self-reported health status, and ‘patient activation’ (knowledge, skills, and confidence to self-manage) [12,13,15]. There is agreement that rural communities are not homogenous and can differ from region to region and rural health disparities require solutions that are tailored and in line with the needs of local communities [20]. Future research needs to appreciate the diverse demographics of these communities by considering the unique characteristics, inequities, and stressors occurring within each rural community [43].

Physical activity has widely been advocated to improve outcomes, quality of life, and overall survival [44,45]. Although, in this study, both urban and rural participants’ engagement with physical activity behaviours was the lowest across all of the health-promoting behaviours. This could be explained by a range of barriers such as treatment-related side effects, self-image and social stigma, inadequate information, lack of time, and cancer-related fatigue [46,47]. Rural participants scored higher on engagement with physical activity behaviours, but this was not statistically significant at our threshold of *p* < 0.01. 

Out of the six subscales, all respondents scored highest in interpersonal relations with a mean value of 2.94 ± 0.58, indicating a good level of social support among the people living with cancer who responded to the questionnaire. That said, the wider literature highlights the detrimental impact that cancer can have on mental health and wellbeing, making communication with close friends and family vital to how well people can cope with the consequences of cancer [48,49]. The qualitative data from the open-ended question also reinforced the importance of non-clinical support to the study participants and how this helped them with ‘moving on’. It should be acknowledged that not everyone will have good levels of social support and people living with cancer who reside in both rural and urban areas have the potential to feel emotionally and physically isolated if they do not have access to social support. Existing cancer survivorship research has shown that cancer survivors use “normality” as a strategy to re-establish and maintain personal identity [50,51]. The idea or act of ‘being normal’ is likely to differ on an individual basis as well as collectively for both rural and urban cancer survivors. Furthermore, what it means to be ‘normal’ will also be influenced by a range of sociocultural factors [50]. Self-management was a prominent theme in the qualitative data for both urban and rural participants and this has been shown to play a significant role in supporting cancer survivors to realign themselves with their own normalities through making adjustments to their lifestyle behaviours following cancer [51]. It is important to understand what self-management practises, including health behaviours, work best for rural and urban cancer survivors so that they can be supported with their recovery as well as reconnecting with their personal idea of ‘normality’.

In this research, rural people living with cancer scored significantly higher than urban participants on the overall HPLP-II as well as on four of the six subscales: health responsibility, nutrition, spiritual growth, and interpersonal relationships. Research has shown that people living in rural areas have high levels of trust and engagement with their local communities and that they tend to be more stoical with regards to their health and less likely to report feelings of distress [6,14,18,52]. When deprivation, age, marital status, and education were controlled for, rural–urban residence was a significant predictor of HPLP-II. In this study, it could be that the rural U.K. setting had a positive impact on cancer survivors’ engagement with health behaviours. This is somewhat at odds with the international literature that mostly points to rurality having a negative impact on cancer experiences and outcomes [1,2,3]. The findings from this study could offer opportunities for conceptual development where the rural environment is used as a tool to facilitate health-promotion and support people with recovery from cancer. Although, it should be noted that, in our sample, urban participants tended to live in more deprived areas when compared with rural participants, which could account for some of our rural–urban differences. To the very best of our knowledge, there are no comparable studies that have used HPLP-II to measure and compare health-promoting behaviours with rural and urban people living with cancer. Globally, there is a lack of consistency or agreement around the definition of ‘rural’ and there needs to be an appreciation that rural people can have unique values and psychosocial needs [2]. Despite similarities, international comparisons between high income countries such as the United Kingdom, the USA, and Australia are increasingly multifaceted and complex [16]. Further confirmatory or refuting research studies are warranted to ascertain the impact of rural–urban residence on health-promoting behaviours both in other parts of the United Kingdom and internationally. These studies could also support our understanding as to whether these findings could be deemed clinically significant and not just statistically significant.

## 5. Limitations

The required sample size of 417 was not reached, which could be down to non-response bias. It could be that the power level was set too high at 95% when performing our sample size calculation, and this could be reduced in future studies using the same outcome with cancer survivors. The findings still highlight statistically significant differences between rural and urban participants, although not quite at the twenty percent difference between scores that we allowed for in our power calculation. Unfortunately, the details of non-responders were not accessible, so it cannot be definitively said as to whether the details of non-responders differed significantly from the final sample. This also made it difficult to apply any weighting techniques to the final dataset to try and align it with the study population. There were missing data on the overall HPLP-II (29.5%) as well as on the six subscales (4.8–10.1%). This could be because of the length of the questionnaire in that it was designed as part of a wider study [12,13,15] with multiple instruments that could have resulted in respondent fatigue. HPLP-II consisted of 52 items on its own and some of the participants felt that the questions were not relevant or appropriate to their specific situation. This was reinforced by the qualitative responses where several participants reported things like *“Due to vascular issues, mobility is impaired, thus questions re exercise are not relevant!”* or *“I have put N/A to several of the dietary choice questions as I am artificially fed* via *a RIG tube”*. Therefore, data for the HPLP-II were thought to be mostly ‘missing not at random’ (MNAR) and thus did not warrant any data substitution or imputations as the responses were likely missing because of the participant’s own individual circumstances, with it not being possible for them to engage with some of the health behaviours given their health needs. Future research should consider the diverse needs and side effects of individuals when collecting data on health behaviours with people who have had a range of different cancers. Future use of HPLP-II could be adapted to include a ‘not applicable’ option where particular questions are not relevant to the participant. Finally, the United Kingdom is diverse geographically and these findings only offer an understanding of post-treatment cancer experiences within the East Midlands region. While these might be generalisable to other U.K. settings, further confirmatory studies in other parts of the United Kingdom and internationally are warranted. 

## 6. Conclusions

This research revealed that U.K. people living with cancer from rural areas have significantly greater engagement with health-promoting behaviours when compared with their urban counterparts. The rural environment has the potential to positively support people living with cancer to engage with health-promoting behaviours following the completion of primary treatment.

## Figures and Tables

**Table 1:** Overall HPLP-II and subscale mean scores.

HPLP-II and subscales	(χ) ± SD	Range	*n*=	% Missing	% Rural Missing	% Urban Missing
Total HPLP-II (52 items)	2.55 ± 0.46	1.38–4.00	160	29.5	48.4	51.6
Health Responsibility (9 items)	2.16 ± 0.53	1.00–4.00	205	9.7	40.0	60.0
Physical Activity (8 items)	2.08 ± 0.73	1.00–4.00	211	7.0	73.3	26.7
Nutrition (9 items)	2.73 ± 0.59	1.00–4.00	216	4.8	30.0	70.0
Spiritual Growth (9 items)	2.72 ± 0.63	1.22–4.00	204	10.1	59.1	40.9
Interpersonal Relations (9 items)	2.94 ± 0.58	1.11–4.00	206	9.2	47.4	52.6
Stress Management (8 items)	2.49 ± 0.55	1.25–4.00	210	7.5	46.7	53.3

Notes: HPLP, health promotion lifestyle profile; (χ), mean; ± SD, standard deviation. Total *n* is different owing to missing data.

**Table 2:** IMD decile scores for rural and urban participants.

Index of Multiple Deprivation Decile	Rural *n* (%) *n* = 103	Urban *n* (%) *n* = 120	Total *n* (%) *n* = 223
Decile 1 10% most deprived	0 (0.0)	10 (8.3)	10 (4.5)
Decile 2 10–20%	0 (0.0)	12 (10.0)	12 (5.4)
Decile 3 20–30%	7 (6.8)	10 (8.3)	17 (7.6)
Decile 4 30–40%	12 (11.7)	11 (9.2)	23 (10.3)
Decile 5 40–50%	17 (16.5)	5 (4.2)	22 (9.9)
Decile 6 50–60%	11 (10.7)	15 (12.5)	26 (11.7)
Decile 7 60–70%	14 (13.6)	16 (13.3)	30 (13.5)
Decile 8 70–80%	17 (16.5)	16 (13.3)	33 (14.8)
Decile 9 80–90%	19 (18.4)	14 (11.7)	33 (14.8)
Decile 10 10% least deprived	6 (5.8)	11 (9.2)	17 (7.6)

Notes: missing data owing to *n* = 4 participants not providing their postcode.

**Table 3:** HPLP II: rural–urban comparison.

	Overall HPLP-II	Health Responsibility	Physical Activity	Nutrition	Spiritual Growth	Interpersonal Relationships	Stress Management
Residence							
*Rural*	2.69 (0.44)	2.27 (0.51)	2.21 (0.71)	2.88 (0.53)	2.86 (0.60)	3.10 (0.57)	2.51 (0.55)
*n*=	72	95	92	100	90	94	96
*Urban*	2.41 (0.42)	2.04 (0.50)	1.98 (0.71)	2.59 (0.60)	2.60 (0.64)	2.80 (0.55)	2.46 (0.53)
*n*=	87	108	116	113	111	110	112
T value	4.122	3.241	2.256	3.829	2.919	3.818	0.740
MD	0.28	0.23	0.22	0.30	0.26	0.30	0.05
95% CI	0.14,0.42	0.09,0.37	0.02,0.42	0.14,0.45	0.08,0.43	0.14,0.45	−0.09,0.20
*p*	0.001	0.001	0.025	0.001	0.004	0.001	0.460

Notes: The values are expressed as means (SD) and independent samples *t*-tests were conducted. MD denotes the mean difference between groups. CI represents the 95% confidence interval.

**Table 4:** HPLP II: multiple linear model of predictors of HPLP-II.

	HPLP-II				
	B	SE B	β	*t*	*p*
Constant	2.367418 (1.929,2.805)	0.222	-	10.679	<0.001
Rural-Urban	0.184 (0.046,0.322)	0.070	0.202	2.639	0.009
IMD Decile	0.029 (0.003,0.055)	0.013	0.165	2.200	0.029
Age	−0.004 (−0.010,0.001)	0.003	−0.111	−1.523	0.130
Marital Status	0.178 (0.017,0.340)	0.082	0.168	2.186	0.030
Qualifications	0.178 (0.039,0.317)	0.071	0.188	2.523	0.013
R_2_	0.23				
Adjusted R_2_	0.20				

Notes. Figures in brackets refer to 95% confidence intervals. Outcome variable: 52 item HPLP-II rated 1–4. Independent variable coding: rural–urban (0 = urban; 1 = rural), IMD decile (1 = 10% most deprived to 10 = 10% least deprived), age (in years), marital status (0 = widowed/single/divorced; 1 = married/civil partnership), and qualifications (0 = lower than degree; 1 = degree or higher).

## Data Availability

The data presented in this study are available upon request from the corresponding author.
